# *Streptococcus suis* infection as an emerging zoonotic threat in Brazil: a One Health-based review

**DOI:** 10.1590/S1678-9946202668019

**Published:** 2026-02-16

**Authors:** Luís Arthur Brasil Gadelha Farias, Osvaldo Mariano Viana, Ednaldo Pereira Lima, Antonio Gutierry Neves Dantas de Melo, Isabela Melo Pontes, Bruna Batista Schmid Gonçalves, Helena Pinheiro Benevides, Susana Pereira de Oliveira Maia, Jorge Luiz Nobre Rodrigues, Lauro Vieira Perdigão

**Affiliations:** 1 Universidade de São Paulo, Faculdade de Medicina, Hospital das Clínicas, Departamento de Moléstias Infecciosas, São Paulo, São Paulo, Brazil; 2 Hospital São José de Doenças Infecciosas, Fortaleza, Ceará, Brazil; 3 Universidade Federal do Ceará, Faculdade de Medicina, Departamento de Saúde Pública, Fortaleza, Ceará, Brazil; 4 Centro Universitário Christus, Fortaleza, Ceará, Brazil; 5 Universidade Federal do Ceará, Faculdade de Medicina, Departamento de Clínica Médica, Fortaleza, Ceará, Brazil; 6 Universidade Federal Rural do Semiárido, Departamento de Medicina Veterinária, Mossoró, Rio Grande do Norte, Brazil

**Keywords:** Streptococcus suis, Zoonosis, Emerging diseases, One Health

## Abstract

*Streptococcus suis* infection is an emerging zoonotic pathogen of growing concern in Brazil, particularly in the Northeast—a region lacking swine-focused surveillance. Although human contamination remains rare, they have been increasingly reported among individuals exposed to pigs or pork products, and most commonly present as central nervous system infections. Diagnostic challenges persist, especially related to culture-based methods, highlighting the need for advanced molecular tools like polymerase chain reaction and metagenomic Next-Generation Sequencing. Veterinary data reveal a high diversity of serotypes and concerning rates of antimicrobial resistance. These studies remain scarce in regions with reports of human infection. This review highlights the clinical, epidemiological, and microbiological aspects of *S. suis* in Brazil and underscores the importance of One Health approaches to enhance detection and prevention.

## INTRODUCTION

Among zoonotic bacterial pathogens, *Streptococcus suis* represents a recent emerging challenge in Brazil^1^. Primarily an animal pathogen responsible for significant infections in the swine industry, it occasionally infects humans, causing endocarditis, pneumonia, sepsis, pyogenic arthritis, and meningitis, among other diseases, with most cases reported in Southeast Asia^1^. In humans, the most frequent symptoms include headache, fever, neck stiffness, and skin lesions related to contact with pigs^1^. It can cause human meningitis which may evolve to death or leave permanent sequelae, most commonly bilateral sensorineural hearing loss^2^. In pigs, under certain conditions, *S. suis* can cross mucosal barriers and enter the bloodstream, spreading to various organs such as the joints and the central nervous system (CNS), leading to systemic infection^3^.

*S. suis* is a Gram-positive, facultative anaerobic bacterium with an ovoid to round shape, typically arranged in pairs or short chains, and exhibits α-hemolysis on sheep blood agar^4^. Its polysaccharide capsule is a major virulence factor, facilitating evasion of host immune defenses, particularly phagocytosis^4,5^. It can be isolated from affected tissues and identified based on biochemical and morphological characteristics^5,6^. Biochemical analysis often reveals a profile consistent with catalase-negative, Voges–Proskauer-negative, and α-hemolytic characteristics on sheep blood agar^6^. The organism typically ferments trehalose, salicin, and sucrose, but not inositol or sorbitol. It is generally positive for leucine aminopeptidase, negative for bile esculin hydrolysis, and non-motile. Traditional identification may include optochin resistance and growth in 6.5% NaCl to differentiate *S. suis* from other streptococcal species^6^.

Although inexpensive and widely accessible, the culture-based approach often lacks the accuracy needed to reliably differentiate *S. suis* serotypes and therefore commonly requires complementary diagnostic methods^6^. Specificity is further limited by phenotypic overlap with other *Streptococcus* species. Other limitations include prolonged turnaround times and modest positivity rates^6^. Serotyping of *S. suis* strains is typically performed by detecting capsular polysaccharide–specific antigens via coagglutination or multiplex PCR assays targeting the capsule synthesis gene cluster^7^. Currently, 29 serotypes of *S. suis* are recognized (serotypes 1 to 34 and 1/2, the latter referring to a variant of serotype 1 that shares antigenic features with serotype 2)^7^, which are distinguished by differences in their capsular polysaccharide antigens. Serotype 2 is the one most frequently associated with clinical infections in both pigs and humans^7^.

Notably, reports of *S. suis* infection are increasing in non-endemic regions outside Asia, including South America, with documented cases particularly concentrated in northeastern Brazil^2,8,9^ despite the country being the fourth-largest pork producer globally^10^. Between 2020 and 2025, cases of *S. suis* meningitis were reported in states like Bahia and Ceara, with ongoing reports by specialized meningitis surveillance groups^2,9^. However, veterinary epidemiological studies on swine production in northeastern Brazil are limited, and the etiological factors contributing to this regional distribution remain poorly elucidated, despite the predominance of human cases in this area^9,10^.

Zoonotic diseases remain under-researched, with limited studies addressing this issue, and the continuous emergence of cases highlights a critical knowledge gap and reinforces the need to prioritize research aimed at achieving a more comprehensive understanding of this phenomenon. Herein, we present a review of the clinical, epidemiological, and veterinary aspects of *S. suis* infection in swine and human cases reported in Brazil. Additionally, we describe two recently diagnosed human *S. suis* infections from northeastern Brazil.

## MATERIALS AND METHODS

The primary question guiding this review was "What is the current epidemiological situation of *S. suis* infections in humans and swine in Brazil?" To address this question, a comprehensive literature search was conducted using the following primary databases: MEDLINE^®^ (via PubMed), LILACS, and Google Scholar, covering the period from January 1970 to October 2025 and including the following terms to retrieve relevant articles: "*Streptococcus suis*" AND ("Pigs" OR "Swine" OR "Swine Infection") OR ("Brazil" AND ("Meningitis" OR "Human infection").

The review only included articles published in English, Spanish, and Portuguese. Information were compiled from previously published, peer-reviewed studies to describe the clinical, epidemiological, and veterinary aspects of *S. suis* infections in Brazil. Inclusion criteria encompassed descriptive cross-sectional studies, observational studies, serotyping reports, outbreak investigations, and descriptive prevalence studies. Case reports, case series, and letters were included when addressing human or swine *S. suis* infection in Brazil. We also reviewed the reference lists of all retrieved articles to identify additional relevant primary sources that might have been missed in the initial search strategy, using a manual snowballing approach. The extracted data were organized into four thematic categories: (1) Epidemiology – *S. suis* outbreaks in Brazilian farms; (2) *S. suis* in pigs – Brazilian data; (3) Human *S. suis* cases in Brazil; and (4) One Health aspects and control measures.

Two newly identified human cases of *S. suis* infection were incorporated into this review. Clinical information was obtained by retrospective evaluation of medical records from patients hospitalized in October 2022 and September 2025 at Sao Jose Hospital of Infectious Diseases (HSJ), a regional referral center for infectious diseases in northeastern Brazil.

### Ethics

This research is part of a retrospective cohort study approved by the HSJ Research Ethics Committee (protocol Nº 7.529.553; CAAE: 52811521.7.0000.5044). Both patients of the newly identified cases provided informed consent for publication of their information.

## RESULTS

Bibliographic search initially identified 9,210 studies which were screened by title and abstract, reducing the selection to 32 articles reporting Brazilian data. Following a full-text analysis, 25 articles met the inclusion criteria. Studies were published in English (n = 18, 72%) and Portuguese (n = 7, 28%). Analyzing the publications per year, the number of articles increased over time, with one in the 70s, two in the 80s, one in the 90s, ten between 2000 and 2018, and an apparent increase in publications in the last five years compared with the previous two decades: 2019 (n = 1), followed by 2020 (n =3), 2021 (n = 1), 2022 (n = 3), 2023 (n = 1), 2024 (n = 2). Studies were divided into human infection cases (n = 4) and carriage (n = 1), epidemiological and prevalence studies (n = 11), swine infection case series (n = 3), focus on genetic analysis, serotyping and antimicrobial resistance (n = 5), and a swine case report (n = 1).

### Epidemiology – *S. suis* outbreaks in Brazilian farms

**Figure 1:**
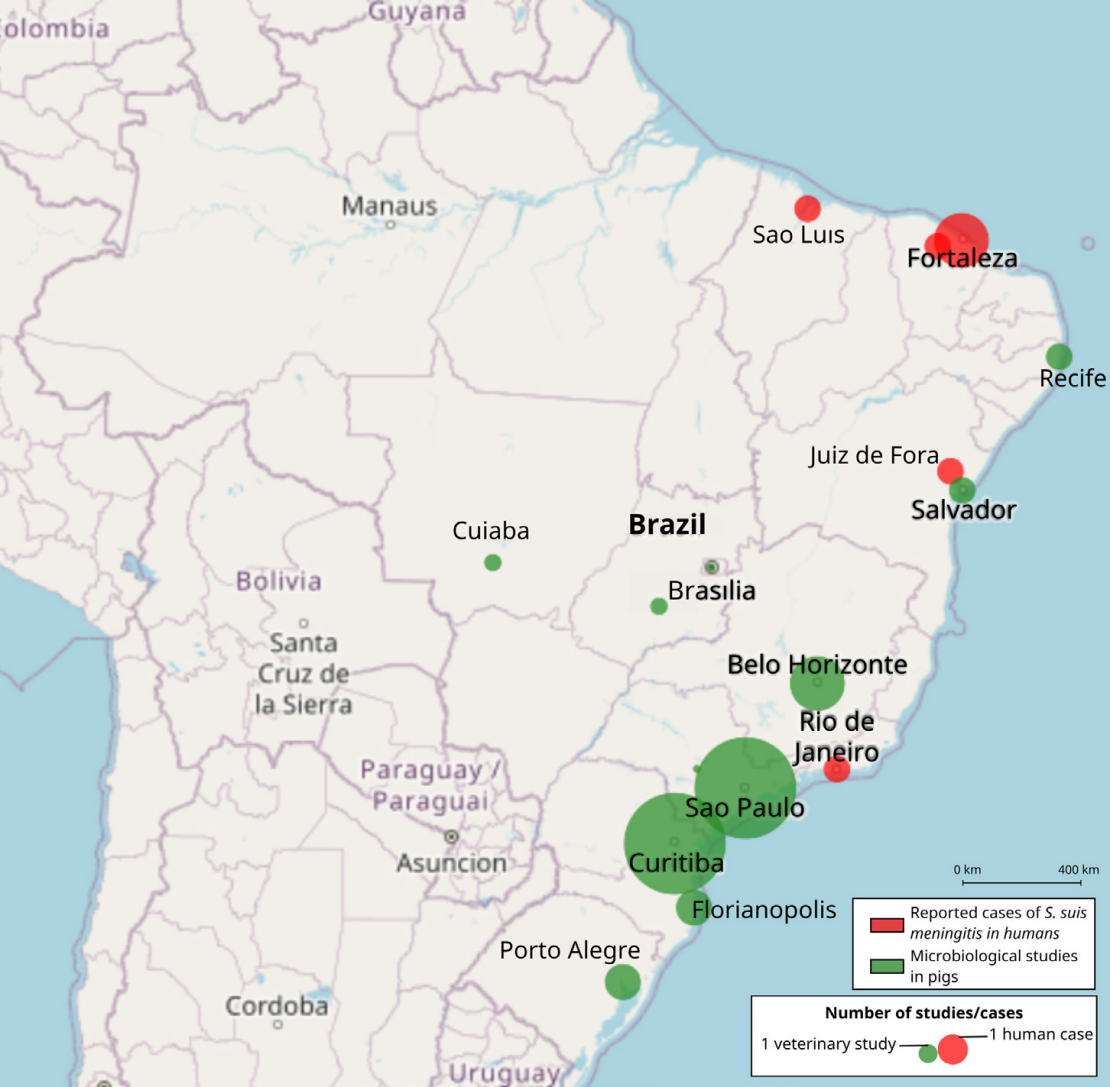
Reported cases of *S. suis* human meningitis in Brazil (red) vs. states with published studies on microbiological aspects of *S. suis* infection in pigs (green).

Most documented *S. suis* infection outbreaks in Brazil have occurred in swine, with no human outbreaks reported to date—only individual case reports^2,9^. The main Brazilian regions affected by *S. suis* outbreaks in swine include Parana, Sao Paulo, Minas Gerais, Santa Catarina States, and the Distrito Federal11. A total of 18 studies were used to better describe the epidemiology of swine outbreaks in Brazil (Table 1). Researched identified 6 (50%) outbreak investigations, most occurring within Brazil's South-Southeast axis (Figure 1). Serotype 2 was the most commonly identified isolate. Other serotypes implicated in the disease included 1, 2, 1/2, 3, 4, 5, 1/14, 6, 7, 8, 9, 10, 14, 18, 28, and 27^11-19^. One recorded outbreak in Parana involved 30 nursery farms, 10 of which had confirmed *S. suis* infection caused by serotype 9^19^.

**Table 1:** Summary of *S. suis* reports, prevalence studies, isolates and outbreak investigations in pigs across Brazil

Article	Year of data collection	Study type	Outbreak investigation	State	Major serotype	Diagnostic methods	Main findings	Affected animals and farms (n)
Diseased Swine								
Clifton-Hadley^25^	1984	Descriptive cross-sectional study	Yes	Sao Paulo, Minas Gerais, Rio de Janeiro	N/A	Culture and biochemical tests	Pyogenic Arthritis in 107 pigs. Three of them with *Streptococcus* Lancefield group R from Brazil in 1977	3 pigs/33 farms
Farinha *et al*.^12^	1981	Case series	Yes	Sao Paulo	Serotype 2	Culture and biochemical tests	Five strains from dead piglets tested were positive for *S.suis* serotype 2	5 piglets
Garcia and Schonhofen *et al*.^13^	1988	Case series	Yes	Parana	Serotype 2	Culture and biochemical tests	Nine pigs developed neurological signs; three died within five days and tested positive for *S. suis* serotype 2	3 piglets
Martinez *et al*.^23^	2003	Descriptive cross-sectional study	No	Sao Paulo, Minas Gerais, Parana	Serotype 2, followed by serotypes 3, 7, 1 and 14	Tissue swabs, culture, biochemical tests, API Strep 20, serotyping via co-agglutination	58.8% were serotype 2; Serotype 2 strains showed a predominantly clonal pattern with an atypical virulence-associated phenotype (MRP+, EF, suilysin+); First large-scale isolation and serotyping in Brazil	51 isolates from sick pigs; >50% of farms affected
Calderaro *et al*.^17^	2004	Descriptive cross-sectional study	Yes	Sao Paulo, Santa Catarina, Parana, Pernambuco, Bahia, Minas Gerais, Goias, Rio Grande do Sul	Serotype 2	PCR and biochemical tests	All samples were serotype 2; 49.5% from São Paulo; 66.9% CNS isolates	133 pigs/88 farms
Costa *et al*.^24^	2005	Experimental laboratory analysis	No	N/A	Serotype 2, followed by serotypes 14, 9, 7, 11, 1, 8, ½, 3, 5, 6 and 10	Culture and serotyping	110 samples were analyzed; 38.2% was the most prevalent; followed by 9.1% serotype 14 and 6.4% serotype 9; 41.9% were lethal to mice	110 isolates
Doto *et al.* ^18^	2016	Retrospective genetic diversity analysis	No	Sao Paulo, Santa Catarina, Parana, Pernambuco Bahia, Minas Gerais, Rio Grande do Sul	Serotype 2	PCR, PFGE typing, SE-AFLP	43.6% virulence genotype were mrp+/epf+/sly+; 62.7% CNS isolates; Eight genotypes were identified, including strains with EF gene variants	103 pigs/ 88 farms
Matajira *et al*.^11^	2019	Retrospective laboratory-based observational study	No	Sao Paulo, Santa Catarina, Parana, Pernambuco, Bahia, Minas Gerais, Goias, Rio Grande do Sul	Serotypes 2 and followed by 3, 7, 1/14, 6, 8, 18, 28, and 27	PFGE typing, capsular, virulence, and antimicrobial resistance profiling	215 strains from 2001-2016; 86% serotype 2/½; 49.3% CNS isolates	215 pigs
Magalhães *et al*.^27^								
	2020	Case report	No	Sao Paulo	N/A	Culture and biochemical tests	A four-year-old, crossbred male pig, with *S. suis* orchitis, from a private property, evolved to death	1 pig
Hammerschmitt *et al*.^19^	2022	Descriptive cross-sectional Study	Yes	Parana	Serotype 9	Culture, Gram staining, and multiplex PCR	30 nursery farms, 10 showed severe *S. suis* associated diseases	10 nursery farms
Almeida^15^	2022	Case series	No	Federal District	Serotype 2	Culture, Gram staining, and serology	Five pigs with neurological signs; 60% CNS isolates and serotype 2.	5 piglets/1 nursery farm
Coldebella *et al*.^14^	2023	Descriptive cross-sectional Study	Yes	Santa Catarina	N/A	Culture and Gram staining	6.32% morbidity; 2.8% mortality in piglets aged 45–55 days; Swines with arthritis and CNS infection	1942 piglets
Healthy Swine								
Barcellos *et al.* ^20^	1995	Descriptive observational study	No	Rio Grande do Sul	Serotype 2	Culture, biochemical tests, and indirect immunofluorescence	Among 239 samples, 81 isolates were classified as serotype 2; 46% were positive by indirect immunofluorescence	81 pigs
Bosco *et al*.^22^	2000	Descriptive cross-sectional study	No	Sao Paulo (Botucatu city)	Serotype 2	Tonsil swab, culture, biochemical tests	10.3% prevalence of serotype II in piglets; High resistance to tetracycline and trimethoprim–sulfamethoxazole; high sensitivity to chloramphenicol, cephalotin, ampicillin, and penicillin	34 piglets/7 farms
Lara *et al*.^21^	2007	Descriptive cross-sectional study	No	Santa Catarina	Serotype 2	Culture	34 herds of animals were analyzed, with 19 testing positive; 27.36% prevalence of serotype 2	340 pigs
Faria *et al*.^16^	2009	Descriptive cross-sectional study	No	Mato Grosso	Serotype 2	PCR	58.7% tested positive for *S. suis*; 23.4% were identified as serotype 2	201 pigs

N/A = Not available data; API Strep 20 = Analytical profile index – Strep 20; CNS = Central nervous system; PCR = Polymerase chain reaction; PFGE = Pulsed-field gel electrophoresis; SE-AFLP = Single-enzyme amplified fragment length polymorphism; IHC = Immunohistochemistry.

Outbreaks due to *S. suis* infection in piglets have been reported since the 1980s in Sao Paulo, Minas Gerais, Rio de Janeiro, and Parana States^12,13^. The first two series, reported in 1981 and 1988, documented outbreaks in piglets involving three and nine animals, respectively^12,13^. At that time, diagnosis relied on brain tissue culture and conventional biochemical testing, which identified serotype 2^12,13^. The largest and most recent outbreak recorded in Brazil occurred in the Santa Catarina State. Among 4,733 piglets, 1,942 presented clinical signs of infection^14^. Six animals evolved to death and underwent necropsy, with tissue samples collected for bacteriological culture and molecular biology analyses, which confirmed *S. suis* as the causative agent^14^.

In the Midwest, four studies were conducted^15,16-18^. PCR-based prevalence studies on 201 slaughtered healthy pigs from Mato Grosso detected serotype 2 presence at levels higher than the average reported in the literature^15^. Calderaro *et al*.^17^ observed lower genotypic diversity in Goias compared to Sao Paulo and Santa Catarina. Although the comprehensive study by Matajira *et al*.^11^ includes strains from Goias, it does not provide sufficient data to discuss the specific characteristics of *S. suis* in that state. Data regarding *S. suis* infection in swine in Northeastern Brazil is scarce, with reports only from Bahia and Pernambuco^15^. States with documented human cases like Ceara and Bahia have not reported frequent *S. suis* identification or outbreaks in swine (Figure 1)^2,9^. In a study involving 215 strains of *S. suis* focusing on serotyping identification, Matajira *et al*.^11^ included data from Bahia and Pernambuco with an emphasis on CNS isolates. A similar study encompassing 113 *S. suis* strains from 103 pigs also included data from Bahia and Pernambuco to better define the main serotypes in Brazil^11,18^. However, neither study specify the number of samples submitted from these states or the serotypes identified by state^15,18^. No data on *S. suis* infection in swine from Northern Brazil were found.

Four studies focusing on *S. suis* prevalence data among healthy swine found that prevalence in Brazil ranges from 10.27% to 55.88%^20-24^. Lara *et al*.^21^, who analyzed 34 samples collected from multiple farms in Santa Catarina, reported the highest prevalence. Among these samples, 19 tested positive for *S. suis* serotype 2, resulting in a prevalence of 55.88%.

Many factors may explain the resurgence of *S. suis* disease outbreaks among swine in Brazil. Clinical reports from Brazilian farms consistently describe outbreaks affecting pigs under physiological or immunological stress, particularly during the nursery and grower phases, when meningitis, septicemia, arthritis, and pneumonia are most frequently observed^17,19^. These outbreaks are strongly influenced by individual animal factors that predispose pigs to bacterial invasion and systemic disease^15,20^. Many Brazilian outbreaks in swine are related to stressors like weaning, transport, mixing of litters, and inadequate colostral immunity, which are repeatedly associated with increased susceptibility as these conditions impair mucosal defenses and facilitate bacterial spread from the tonsils, a common site of asymptomatic carriage^11-19^. Additional factors contributing to Brazilian outbreaks include co-infections with viruses or *Mycoplasma* spp. and environmental or management deficiencies that compromise immune competence, thereby increasing individual vulnerability and enabling the expression of virulence determinants such as suilysin, MRP, and EF^15,17,20,23,24^. Moreover, the genetic diversity of circulating *S. suis* serotypes—including serotypes 2 and 9—may shape disease emergence, as some strains exhibit greater pathogenic potential, exemplified by the serotype 9 outbreak reported in Parana^18,19,23,24^.

### *S. suis* in pigs – Brazilian data

*S. suis* infection in swine in Brazil was first reported in 1977 during an investigation of synovial samples from cases of bacterial arthritis across 33 farms in Minas Gerais, Sao Paulo, and Rio de Janeiro^25^. *S. suis* was identified in three samples and was initially classified as belonging to Lancefield Group D, but was later found to be very similar to Group R^12,13^. During the 1980s, reports of *S. suis* meningitis in swine increased in Brazil^12,13^. Meningoencephalitis caused by this microorganism in pigs was first described in 198112. Eighteen strains of alpha-haemolytic streptococci were obtained during 1979 from the brains of piglets 6–10 weeks old with nervous disorders, and another 10 strains during 1980^12^. Five of these 10 strains (sent to Atlanta, Georgia, USA for typing) were *S. suis* serotype 2, at that time known as group R^12^. In 1988, an outbreak of neurological symptoms was reported in nine suckling pigs. Of these, three died within a median of five days and six recovered after treatment with oxytetracycline, some of which developed neurological sequelae and reduced responsiveness. *S. suis* serotype 2 was identified through culture and biochemical testing^13^.

A series of two piglets from Parana with clinical meningitis from the same farm revealed atypical neurological signs related to an *S. suis* CNS infection such as ataxia, opisthotonus, paddling movements^26^. The 14-day-old piglet survived after treatment with procaine benzylpenicillin for three days, whereas the 30-day-old piglet died despite therapy with amoxicillin and gentamicin^26^. According to Matajira *et al*.^11^, meningitis constitutes the most frequent clinical manifestation in swine, followed by polyarthritis, endocarditis, pneumonia, septicemia, polyserositis, and sudden death. Rarely, it can also present as endometritis or miscarriage^11^. During the largest *S. suis* outbreak in piglets in Santa Catarina, necropsies performed on six pigs with fatal outcomes revealed suppurative polyarthritis in five cases and meningitis in one^14^. Other case reports from Brazil highlight other atypical manifestations such as orchitis on a four-year-old, crossbred male pig with orchitis from a private property, who was later confirmed for *S. suis* etiology^27^. Overall, post-weaning piglets aged 5 to 10 weeks seem particularly more susceptible to clinical infection, with common clinical signs including fever, anorexia, neurological symptoms, lameness, and death being frequently described in Brazilian studies, mainly in Southeastern Brazil (Figure 1)^12,24,27,28^.

**Figure 2:**
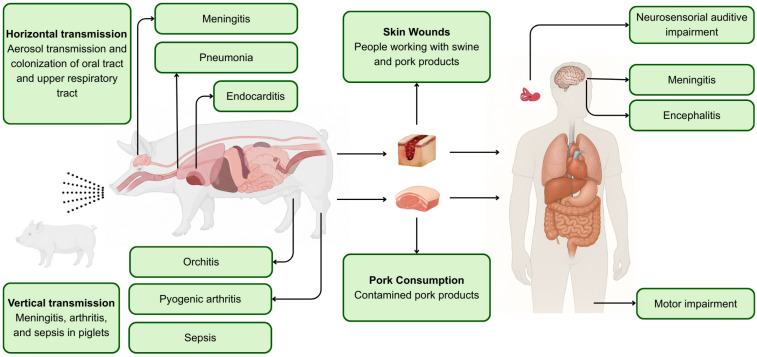
Main transmission routes and clinical manifestations of *S. suis* infection in swine and humans in Brazil. Horizontal and vertical transmission in pigs lead to meningitis, pneumonia, arthritis, and sepsis. Human infection occurs primarily through skin wounds or consumption of contaminated pork, resulting in meningitis, encephalitis, neurosensory hearing loss, and motor impairment.

Brazilian data suggests a combination of individual, environmental, and management-related risk factors that predispose pigs to systemic disease^12,13,26-28^. Most clinical reports describe swine under physiological stress—particularly during the nursery, growing, and finishing phases—as being at higher risk, with weaning, mixing of litters, transport, and failures in passive immunity transfer identified as major contributors to disease onset^28,29^. Histopathological evaluations commonly document suppurative CNS inflammation, vasculitis, fibrinosuppurative exudate, and bacterial emboli—hallmarks aligned with the known capacity of the pathogen to cross the blood–brain barrier and trigger intense neutrophilic responses (Figure 2)^12,19,28^.

### Antimicrobial resistance in swine

Four studies directly investigate antimicrobial resistance in swine in Brazil^10,11,22,29^. Antimicrobial resistance is an increasingly significant concern among *S. suis* isolates in swine. Brazilian veterinary studies on the antimicrobial susceptibility of *S. suis* are limited and primarily concentrated in Sao Paulo State^10,11^. Antimicrobial susceptibility testing of 260 *Streptococcus suis* isolates from clinically healthy swine in Sao Paulo revealed widespread multidrug resistance, with over 99% of strains resistant to at least three antimicrobial classes. These findings are comparable to, or even more concerning than, those reported internationally^10^. A high resistance rate was observed for tetracyclines (97.69%), sulfonamides/trimethoprim (100%), macrolides (46.54%), and lincosamides (84.61%)^10^. However, β-lactams (particularly ceftiofur and cephalexin) showed good activity (n = 260)^10^. Notably, the study did not identify meaningful resistance differences among farms within the sampled region^10^.

Matajira *et al*.^11^ characterized 215 *S. suis* isolates collected from pigs across several Brazilian states between 2001 and 2016, finding a predominance of serotype 2 and ½ (86%) by molecular serotyping. They identified nine distinct antimicrobial resistance profiles, with some strains resistant to as many as eight antimicrobial classes. Multidrug resistance was particularly frequent, affecting 72.1% of the isolates, and resistance increased in strains isolated after 200911. Nonetheless, β-lactams (e.g., penicillin, ceftiofur) and florfenicol remained among the most effective agents^11^. In an earlier study published in 2008, amoxicillin and florfenicol showed the greatest antimicrobial activity, with 90% of *S. suis* isolates (n = 70) exhibiting susceptibility at the established minimal inhibitory concentration (MIC) levels^29^.

### *S. suis* human cases in Brazil

Despite the high prevalence of *S. suis* in pigs (with the bacterium detected in 58.8% of animals on farms in Sao Paulo, Minas Gerais, and Parana^11^, particularly serotypes 2 and 9) in Brazil, cases of *S. suis* meningitis in humans are rare. In 2014, a study conducted in three pig farms in Sao Paulo tested 28 farm workers for *S. suis* carriage, confirming the first case in the country of a human carrier, without any signs and symptoms at the time^30^.

Human cases of *S. suis* meningitis in Brazil remain rarely reported in the literature, primarily as isolated case reports or small case series^2,9,31,32^. To date, five human cases have been documented in the country, as reported in four peer-reviewed publications up to 2020^2,9,31,32^. No cases had been recorded before 2020. Herein, we describe the clinical and laboratory characteristics of each previously reported case and include two additional cases recorded at our infectious diseases center between 2022 and 2025, summarizing key clinical findings, exposure risks, laboratory data, and outcomes.

Most patients were male (5:2 ratio) with a median age of 63.1 years, from Northeast Brazil, specifically Ceara State. Exposure risk was identified in five out of seven individuals (71.4%), four through occupational exposure and one through pork consumption. In recent years, three cases were reported in Ceara and Bahia, all with detailed epidemiological histories involving pig farming and direct contact with swine^2,9,31,32^. Most patients (5/7; 71.4%) presented with neutrophilic pleocytosis, elevated protein levels, and decreased glucose in the CSF analysis. Gram staining revealed Gram-positive cocci in all cases. Diagnosis was predominantly established by CSF culture (6/7; 86.7%). Serotyping was performed in four cases, identifying two *S. suis* serotype 2 isolates (Ceara and Bahia) and one serotype 1 isolate (Ceara)^2,9^. No deaths were reported; however, three patients (42.8%) developed sensorineural hearing loss, and two (28.6%) presented significant motor deficits.

### Case 1

The first case reported was documented in 2020, in Rio de Janeiro, in an 82-year-old Brazilian man, without previous comorbidities, three days after pork consumption. The patient was admitted in September 2019 with a clinical picture of meningitis (fever, nuchal pain, and headache). Lumbar puncture revealed an opening pressure of 29 mmHg, a cloudy appearance, a white blood cell (WBC) count of 2,133/µL with 75% neutrophils, total protein of 126 mg/dL, and glucose of 66 mg/dL. Gram stain revealed rare cocci, and Gram-positive diplococci were observed. CSF culture was negative. Blood samples were analyzed using matrix-assisted laser desorption ionization-time of flight mass spectrometry (MALDI-TOF MS), confirming identification of *S. suis*. The strain was susceptible to penicillin and ceftriaxone (MIC < 0.06 μg/mL and < 0.12 μg/mL). Serotype identification was not achieved. He was initially treated with ceftriaxone, vancomycin, and acyclovir, which were later de-escalated to ceftriaxone alone at 2 g every 12 h. He required intensive care unit (ICU) admission but had a favorable clinical progression. He was discharged in good condition, and no data regarding sequelae were available. The authors believe that the *S. suis* infection was possibly acquired through pork consumption, without any prior direct contact with pigs^31^.

### Case 2

The second case was documented in 2021, in Ceara State, northeastern Brazil. A 60-year-old male butcher who worked in a pig slaughterhouse, with previous alcoholism and chronic obstructive pulmonary disease, presented with fever, headache, and meningeal signs. Curiously, he already presented hypoacusis and tinnitus at symptoms onset. CSF analysis revealed 82 WBC/μL with 48% polymorphonuclear (PMN) leukocytes, glucose of 5 mg/dL, CSF protein of 143 mg/dL and presence of Gram-positive diplococci. CSF culture identified *S. suis* serotype 2, susceptible to ceftriaxone. The patient was treated with ceftriaxone (2 g every 12 h for 14 days) and dexamethasone (4 mg every 6 h for 5 days), achieving complete clinical recovery. Follow-up audiometry, however, revealed bilateral sensorineural hearing loss^2^.

### Case 3

This case was registered simultaneously to case 2, in the same year. A 68-year-old male pig farmer presented with fever, headache, nuchal stiffness, loss of consciousness, and dysarthria. Given the suspicion of meningoencephalitis, computer tomography (CT) of the brain was performed and abnormal and CSF analysis showed glucose levels of 22 mg/dL, a protein concentration of 320 mg/dL, a cell count of 1,322 cells/μL with 44% polymorphonucleocytes, and a Gram stain with no findings. CSF culture identified *S. suis* serotype 1 by VITEK^®^ 2, susceptible to piperacillin-tazobactam (MIC < 0.06 µg/dL). Initial therapy with piperacillin-tazobactam (4.5 g every 6 h for 7 days) was switched to meropenem (1 g every 8 h for 14 days) and polymyxin B (25,000 IU/kg/day for 7 days) due to secondary pulmonary infection with *Pseudomonas aeruginosa*. The patient showed a favorable clinical response and was discharged after nearly one month, with no signs of relapse on follow-up^2^.

### Case 4

A case from Maranhao State, similar to the first documented case in Brazil, was admitted with a classic presentation of acute bacterial meningitis but had no previously known compatible epidemiological history^35^. The patient was a 52-year-old woman with typical meningitis and dizziness that progressed to tinnitus and bilateral hearing loss. CSF analysis showed 160 WBC/μL (32% PMN, 68% mononuclear), protein of 114 mg/dL and glucose of 3 mg/dL. Gram-positive cocci in a single chain were observed, and cultures grew *S. suis*. Serotype identification was not performed. She was treated with ceftriaxone and dexamethasone, with full clinical recovery and no sequelae at discharge. Despite the initial picture with additional auditive symptoms, such as hearing loss, was not documented^32^.

### Case 5

In 2022, another case involving a Brazilian woman from Ceara presenting clinical features compatible with severe acute meningitis and sensory loss, without prior risk or epidemiological factors, was admitted in an infectious diseases hospital. CSF analysis revealed 76 WBC/μL with 54% PMN, glucose of zero mg/dL, CSF protein of 410 mg/dL, adenosine deaminase 17,4 U/L (reference: < 9 U/L) and presence of Gram-positive diplococci. She was treated with ceftriaxone 2g every 12 h, vancomycin 15mg/kg/day every 12 h, and dexamethasone 0,4 mg/kg/day. The patient required ICU admission with invasive mechanical ventilation for eight days. After a 20-day hospitalization, she was discharged without sequelae. Epidemiological investigation revealed no notable association with occupational exposure or pork consumption.

### Case 6

In 2024, a 68-year-old man from Bahia State, northeastern Brazil, with no comorbidities, who works as a pig farmer, was admitted with meningitis. Examination revealed meningeal signs and papilledema. During hospitalization, he developed bilateral hearing loss, balance disturbances, and difficulty walking even with mobility aids. CSF analysis showed 350 WBC/μL, with 89% PMN, CSF protein of 119 mg/dL and glucose of 32 mg/dL. Gram-positive cocci grouped in pairs and short chains were evinced, with culture confirming *S. suis* serotype 2. He was treated with ceftriaxone for 14 days, leading to clinical and laboratory improvement, though motor and auditory deficits persisted at discharge, requiring ongoing rehabilitation. This case provided the first evidence of motor deficits after *S. suis* meningitis in Brazil^9^.

### Case 7

**Figure 3:**
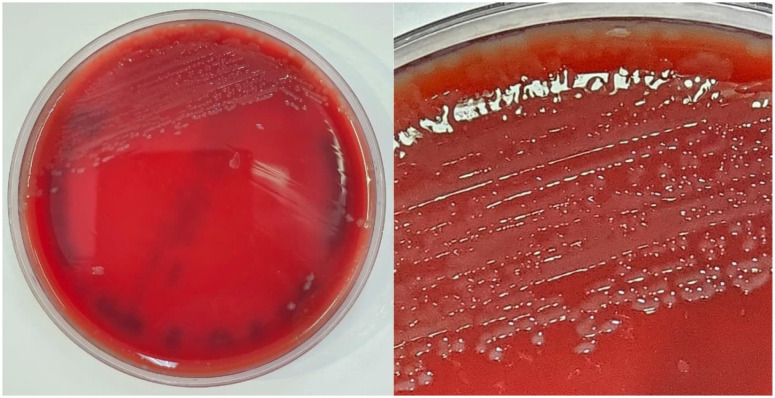
*S. suis* culture on blood agar showing small α-hemolytic colonies after 24 h of incubation.

A 73-year-old male pig farm worker responsible for the swine castration sector was admitted in September 2025 with high fever, headache, and sensory loss. CT of the brain was unremarkable. Lumbar puncture revealed turbid CSF with 7.205 WBC/μL (97% PMN), protein of 712.5 mg/dL, and glucose of 8 mg/dL. CSF lactate was markedly elevated at 115 mg/dL (reference: 9–16 mg/dL). Culture grew *S. suis*, though the serotype was not identified and susceptibility data were unavailable (Figure 3). The patient was initially treated with ampicillin-sulbactam 2g every 6 h and later switched to ceftriaxone 2 g every 12 h. After 21 days of hospitalization, he was discharged with bilateral sensorineural hearing loss and severe motor sequelae, including inability to walk due to limb weakness and ataxia. Cochlear implantation was subsequently performed, resulting in improved hearing.

No human cases have been documented in the medical literature from Northern, Midwestern, or Southern Brazil. Table 2 provides a summary of the key clinical manifestations, main CSF findings, corticosteroids use, therapeutic approaches, sequelae, and clinical outcomes for each case.

**Table 2:** Summary of *S. suis* meningitis cases in Brazil

Article	Year of data collection	Age	Gender	Risk/exposure factor	State	Serotype	Main CSF findings	Clinical syndrome	Diagnostic methods	Treatment*	Corti-costeroids	Outcome	Sequelae
Silva *et al*.^31^	2019	82	Male	Pork ingestion	Rio de Janeiro	N/A	Neutrophilic pleocytosis and Gram-positive diplococci	Meningitis	Blood Cultures, MALDI-TOF-MS	Cfx plus Van and Acyclovir	No	Hospital discharge	N/A
Matos *et al*.^2^	2021	60	Male	Butcher	Ceara	2	Lymphocytic pleocytosis and Gram-positive diplococci	Meningo-encephalitis	CSF culture (VITEK^®^ 2)	Cfx	Yes	Hospital discharge	Bilateral sensorineural hearing loss
Matos *et al*.^2^	2021	68	Male	Pig Farmer	Ceara	1	Neutrophilic pleocytosis and Gram-positive diplococci	Meningitis	CSF culture (VITEK^®^ 2)	Piperacillin-tazobactam, Meropenem**	No	Hospital discharge	No sequelae
Batista and Correia^32^	2022	52	Female	N/A	Maranhao	N/A	Lymphomono-nuclear pleocytosis and Gram-positive diplococci in a single chain	Meningitis	CSF culture	Cfx	Yes	Hospital discharge	No sequelae
Farias *et al*. (this study)	2022	39	Female	unemployed	Ceara	N/A	Neutrophilic pleocytosis and Gram-positive diplococci	Meningo-encephalitis	CSF culture (VITEK^®^ 2)	Cfx plus Van	Yes	Hospital discharge	Bilateral sensorineural hearing loss
Ramos *et al*.^9^	2024	68	Male	Pig Farmer	Bahia	2	Neutrophilic pleocytosis and Gram-positive diplococci in short chains	Meningitis	CSF culture (VITEK^®^ 2), MALDI- TOF-MS	Cfx plus ampicillin	No	Hospital discharge	Bilateral sensorineural hearing loss and Motor deficit
Farias *et al*. (this study)	2025	73	Male	Pig Worker	Ceara	2	Neutrophilic pleocytosis and Gram-positive diplococci	Meningo-encephalitis	CSF culture (VITEK^®^ 2)	Initially ampicillin Later Cfx	No	Hospital discharge	Bilateral sensorineural hearing loss Motor deficit

ADA = adenosine deaminase; Cfx = ceftriaxone; CSF = cerebrospinal fluid; N/A = not available; MALDI-TOF-MS = Matrix-assisted laser desorption/ionization time-of-flight mass spectrometry; PMNs = polymorphonuclear; Van = vancomycin; WBC = white blood cells;

* Ceftriaxone was administered at a dose of 2 g every 12 h; vancomycin at 15 mg/kg/day; acyclovir at 12.5 mg/kg every 8 h; and meropenem at 2 g every 8 h.

** Initially treated with Piperacillin-tazobactan 4,5g every 6 h, and later changed to meropenem at a standard dose.

### Antimicrobial resistance in human infections

Among the seven patients, four responded to ceftriaxone. Some initially received combined therapy with ceftriaxone and vancomycin, likely administered due to an early suspicion of *Streptococcus pneumoniae* infection^2,9,31,32^. MIC susceptibility data were available for three cases, specifically cases 1, 2, and 3, showing susceptibility to penicillin and ceftriaxone (MIC < 0.06 μg/mL and < 0.12 μg/mL), ceftriaxone (MIC < 0.25 µg/mL) and to piperacillin–tazobactam (MIC < 0.06 µg/mL), respectively^2,31^. The sixth patient did not respond to ceftriaxone and, due to the emergency situation, was ultimately treated with a combination of vancomycin and cefepime. Most human data from cases reported in Brazil are based on CSF isolates and usually do not include antimicrobial susceptibility profiles.

### One Health aspects and control measures

*S. suis* infection represents a potential emerging pathogen threat in Brazil and Latin America, affecting human and swine populations. This scenario underscores the urgent need for integrated veterinary and human health approaches^31^. Antimicrobial resistance of *S. suis*, originating in industrial swine reservoirs, is a concerning issue that could potentially result in resistant zoonotic diseases, as already documented in Brazil^10,29^. The prolonged and indiscriminate use of tetracyclines and macrolides in pig farms, leading to breeding grounds for antibacterial resistance, must be regulated and addressed more comprehensively at both scientific and policy levels. Fortunately, antimicrobial resistance surveillance programs in veterinary medicine, such as the European Antimicrobial Resistance Surveillance Network in Veterinary Medicine (EARS-Vet), are being implemented, proposing intercontinental vigilance regarding these potential zoonotic pathogens (or resistant reservoirs)^33^. Similarly, Brazil has created the National Action Plan for Prevention and Control of Antimicrobial Resistance in Agriculture (PAN-BR AGRO)^34^. However, likely due to the relatively new emergence of this pathogen in Latin America, the PAN-BR AGRO document, unlike articles published by the EARS-Vet group, does not yet specifically address *S. suis*. Currently, the Brazilian government, through MAPA (Ministry of Agriculture, Livestock and Supply), coordinates surveillance, enforces reporting of outbreaks, and oversees transport and trade of animals to limit disease spread, but with no specific measures regarding *S. suis* infection^35,36^.

Studies of human *S. suis* cases are limited. A proactive control measure would involve establishing government-funded research (possibly related to PAN-BR AGRO programs) or veterinary health programs to investigate the prevalence, clinical relevance, and antimicrobial resistance of *S. suis* infection in veterinary settings, particularly in industrial swine production, especially in regions with confirmed human cases but lacking published research on swine, such as northeastern Brazil (Figure 1)^34^. While bacterial culture remains a widely used method for pathogen identification in research and monitoring, it is both costly and time-consuming^6,7^. Molecular methods offer excellent tools for veterinary surveillance, but their use is limited in regions such as northeastern and northern Brazil^2,7^. In these areas, *S. suis* is not considered a significant pathogen as it is in Asia, and research resources are less readily available compared to southern and southeastern Brazil^2,9,11-19^. New methods to facilitate surveillance are being developed. Hatrongjit *et al*.^37^, for example, discuss the challenge of lacking a readily available diagnostic tool for this bacterium and propose a gene *ROK*-based multiplex PCR assay capable of distinguishing all 29 *S. suis* serotypes and predicting the pathogenic pathotypes of *S. suis* isolates from humans and pigs in a single assay. Moreover, PCR-based methods can facilitate intensive surveillance of antimicrobial resistance, enabling the mapping of bacterial resistance genes^6,37^.

## DISCUSSION

This review highlights the emergence of *S. suis* infection in humans and pigs in Brazil. Until 2020, no human cases of *S. suis* infection had been reported in the country8. Over the past five years, however, the emergence of human cases raised concerns within the scientific community concerning epidemiology changes and highlights the need for public health measures to prevent further occurrences^2,9,31,32^.

*S. suis* infection represents a global zoonotic concern with striking regional differences in prevalence and clinical presentation. The disease is most prevalent in Southeast Asia, where it constitutes a major public health issue strongly linked to occupational exposure and consumption of raw pork or pig blood^4^. Large outbreaks in China (1998 and 2005) and the increasing number of human cases in Vietnam and Thailand have driven a marked rise in reported infections, with estimated incidence rates of 5–8 cases per million inhabitants and mortality rates ranging from 2% to 4%^4,38,39^. In China, which harbors the world's largest pig population, prevalence of *S. suis* infection in swine averages 40.8%, reaching over 70% in certain provinces with recurrent human outbreaks^38^. In Hong Kong, occupational exposure increases infection risk by over 300-fold compared with the general population^39^. Sporadic cases have been documented in Europe, particularly in the Netherlands, the United Kingdom, Denmark, Germany, and Spain, accounting for roughly 8.5% of global infections^40^. European cases predominantly affect middle-aged men exposed occupationally, presenting mainly as meningitis (83%) and sepsis (68%), with fatality rates of 13–30%^41^. In North America, despite large-scale pork production, only a handful of human infections have been reported in Canada and the United States, possibly reflecting diagnostic underreporting or lower virulence of circulating strains^42,43^. In contrast, South America has recently documented increasing numbers of human and swine infections, with 47 human cases reported between 1995 and 2024—mostly meningitis (85%)—and a case fatality rate of 4%^8^. Cases were registered in Argentina (n = 29), followed by Chile (n = 7), Brazil (n = 5), Uruguay (n = 5), and French Guiana (n = 1)8. Argentina and Brazil account for most recently described cases, paralleling their expanding pork industries and highlighting occupational exposure among farm and abattoir workers as a key risk factor^8^.

Brazil exhibits a pattern similar to that observed in Europe, where most human infections are associated with occupational exposure among pig industry workers and farmers^8,40,41^. In contrast, in several Asian countries—particularly China, Vietnam, and Thailand—the primary risk factor related to the consumption of raw or undercooked pork^4,38,39^. Although South America, including Brazil, has shown a recent increase in identified *S. suis* human cases, the overall incidence in the region remains considerably lower than in Asia and even some European countries^8,38-41^. This discrepancy may reflect not only true epidemiological differences but also variations in surveillance intensity, diagnostic capacity, and reporting practices across regions. Notably, Argentina has shown a similar upward trend, reporting a higher number of cases (n = 29) and more available sequencing data than Brazil^8^. Similarly, serotype 2 predominates in both clinical cases and zoonotic infections worldwide; other serotypes—such as 9 in parts of Europe and 3, 7, and 8 in Asia—are more frequently associated with the disease in specific regions^8,38-41^. Brazil exhibits high *S. suis* serotype diversity, with most human infections linked to serotype 2, although numerous other serotypes have been associated with swine disease (1, 2, 1/2, 3, 4, 5, 1/14, 6, 7, 8, 9, 10, 14, 18, 27, and 28)^11-19^.

*S. suis* epidemiology in Brazil may be changing, and this is possibly related to factors such as the scale of the country's industrial pig farming sector, which currently ranks as the fourth largest pork exporter in the world^44,45^. Working on pig farms exposes rural workers to an increased risk of contracting zoonoses^2^. *S. suis* is a well-known pathogen that forms part of the commensal microbiota of young and adult swine in Brazil, with prevalence studies evincing its presence in tonsil swabs and documenting its involvement in swine disease outbreaks^11-19^. It may be transmitted in swine throughout aerosol (horizontal transmission) or vertical transmission (Figure 2)^1^. Piglets under physiological or immunological stress—especially during the grower phase in nursery farms—may develop invasive *S. suis* disease, a condition already described in Brazilian outbreaks^11-19^. Pig farmers and workers who have daily contact with these animals may acquire the microorganism and progress to clinically significant disease^2,9,31,32^. This cycle and the intrinsic connection between humans, pork production, and swine health underscore the urgent need for integrated veterinary, including veterinary management and outbreak control measures, as well as human health approaches, including molecular and whole-genome sequencing (WGS) investigations1. Strengthening of the pig farming sector in the Brazilian market, particularly in the southern region, has increased biological risks associated with the production process. In this context, workers in direct contact with pigs are susceptible to infection through skin lesions exposed to the animals, accidental bites, accidental exposure of mucous membranes, aerosols, among other routes^44^. Likewise, the growing importance of the pork industry in Brazil also increases the risk of oral transmission via consumption of contaminated and undercooked pork^45^.

Counterintuitively, some reported cases lacked an epidemiological link such as the report from Maranhao and the last case from Ceara^2,32^. This may be related to an unidentified exposure or may highlight the involvement of *S. suis* in other everyday practices not yet recognized as risk factors. The emergence or resurgence of more virulent strains, coupled with increased disease pressure within swine production systems, may also contribute to the observed rise in human cases. However, it remains possible that the true epidemiological incidence has not substantially changed and that the apparent increase in reported cases primarily reflects improved clinical recognition and diagnostic capacity^2,9,31,32^. Moreover, grater awareness among healthcare providers, broader access to molecular diagnostic methods, and strengthened surveillance systems may be identifying infections that previously went unrecognized or were misclassified^5,38^. On the other hand, access to modern diagnostic tools like molecular methods remains limited in northeastern Brazil^2,9,32^. Consequently, the perceived rise in incidence may largely represent improved case ascertainment rather than a genuine shift in transmission dynamics. Additionally, although the total number of documented human cases remains low, the short time frame in which they were detected—mainly after 2020—may still be epidemiologically significant and suggest emerging tendencies^8^.

Currently, Ceara is one of the largest states in Northeastern Brazil with the highest number of reported *S. suis* human cases^2^, all presenting predominantly as CNS infections^2^. The complete absence of reports from pig farming operations in Ceara is a notable finding (Figure 1). Most research on swine diseases in this state has focused on classical swine fever, which reemerged in 2018^46^. Data on classical swine fever abounds, but no data are available regarding other pathogens of human health interest, such as *S. suis*, *Mycoplasma suis*, and others^46^. This disconnect is noteworthy, as a One Health approach is essential to prevent infections in both animals and humans^1,31,33,46^. *S. suis* infections in humans occurring in previously unaffected areas of Brazil should foster research development and stimulate interest in epidemiological surveillance and veterinary microbiology in these regions. Interestingly, other zoonotic CNS infections have emerged in Ceara, possibly related to the advancement of molecular diagnostic methods not available before such as *Streptococcus equi* subsp. *zooepidemicus* meningitis, which affected five individuals in 2019, an outbreak related to unpasteurized milk^47^.

Such contrast between the geographic distribution of human cases and the lack of swine-focused studies indicates that unrecognized porcine circulation may exist in these regions, but available evidence is insufficient to establish any causal relationship. Northeast states, particularly Ceara, lack veterinary laboratories for diagnosing zoonotic pathogens like *S. suis*, which can contribute to infections in humans at risk2. Other contributing factors include the distribution of pig farming, which is most concentrated in the South, Southeast, and Midwest, followed by the Northeast and North^48^. Notably, however, countries like the United States and Canada, despite having a livestock population of over 115 million pigs, report very few human cases of *S. suis* infection^42,43^. This suggests that the mere presence of the infection in pigs is not sufficient; close occupational exposure without appropriate personal protective equipment and infrastructure may be key contributing factors^2,9,39^. Moreover, the high number of human diagnoses in the Northeast may result from Ceara's strong infectious disease surveillance and our group's active retrospective investigation, which identified 4 of the 7 Brazilian cases^2^. Although *S. suis* infection in swine has been known in Brazil since 1977, epidemiological studies and surveillance remain insufficient, mainly in northeastern Brazil^25^.

All documented human infections reported in Brazil have exclusively involved CNS manifested as acute meningitis or meningoencephalitis^2,9,31,32^. We found no data of human infection causing other clinical manifestations such as endocarditis, arthritis, or sepsis in Brazil. Notably, while no fatalities have been reported, a relatively high proportion of patients developed auditory sequelae, affecting over 50% of cases, and, less common, motor impairments, observed in approximately 28% of patients. Similarly, Romania has experienced an emergence of *S.suis* invasive infection in humans, reporting eight cases with very similar patterns. Most cases were male, with diagnosed meningitis (n = 7; 87.5%) and had deafness as a sequelae (n = 4; 50%)^49^. The Romanian series presented endocarditis cases and ataxia as a post-infectious sequelae, which was not observed in Brazil. Comparable to our findings, no fatalities were reported in Romania^49^. The absence of deaths in our study aligns with existing literature, which describes *S. suis* mortality as generally low—approximately 3–4% in meningitis—and higher, up to 10–13%, in invasive forms of the disease^4^. Hearing loss is a well-documented sequelae, and its pathophysiological mechanisms are attributed to intense inflammatory damage within the inner ear, particularly involving the cochlea. The infection triggers cytokine-mediated injury and disruption of the blood–labyrinth barrier, ultimately resulting in sensorineural hearing impairment^2^.

*S. suis* can be isolated from affected tissues and identified by biochemical and morphological features using low-cost culture methods; however, these approaches often fail to accurately differentiate serotypes and may confuse *S. suis* with closely related species such as *S. mitis* and *S. viridans*, leading to underestimation of its true prevalence^50^. In Ceara, all four registered cases were diagnosed using VITEK^®^ 2^2^. A recent review highlights the potential for misidentification by automated systems like VITEK^®^ 2, as *S. suis* may be incorrectly classified as other streptococcal species, contributing to the underreporting of human cases^50^. In human outbreak settings, VITEK^®^ 2 has been used to identify *S. suis* with relatively high probability scores; however, these results typically require confirmation by PCR or mNGS^51,52^. Molecular techniques, particularly PCR, have become the preferred diagnostic tools due to their superior sensitivity, specificity, and rapid turnaround, enabling precise serotype differentiation and reliable detection even after antimicrobial therapy^37,52^. More recently, advanced molecular tools, including metagenomic, Enzyme-Linked Immunosorbent Assay (ELISA), immunosensor-based assays, and MALDI-TOF MS, have further improved diagnostic accuracy^53-55^. Studies show that metagenomic Next-Generation Sequencing (mNGS) outperforms traditional culture methods in detecting *S. suis* in cerebrospinal fluid, whereas MALDI-TOF MS provides a rapid, high-throughput identification platform whose reliability continues to increase as spectral databases expand, reinforcing its potential as a frontline diagnostic approach when complemented by confirmatory molecular analyses^53-55^. A key limitation of molecular methods lies in their restricted availability and high cost in developing countries such as Brazil.

One factor possibly contributing to the discrepancies in *S. suis* identification between swine and human cases is the distinct availability of diagnostic tools between Brazilian regions. Advances in diagnostic techniques may have facilitated human case detection; however, the availability of molecular methods for diagnosing swine cases remains limited in Brazil^5,6,53-55^. In contrast, laboratories in Asian countries—where the pathogen is endemic and cases are relatively frequent—are better equipped and prepared for early detection of *S. suis* infection^38,39,53-55^. Another aspect is the absence of reports including WGS of *S. suis* isolates originating from Brazil, which severely limits our ability to assess the genomic diversity, clonal lineages, and virulence factors of Brazilian strains in a global context^8^. One of the first studies to map the *S. suis* genome by WGS was a comparative analysis of strains P1/7 and 89-1591, which revealed their genomic organization and average nucleotide identity (ANI)^56^. The lack of WGS data from Brazil also hinders implementing ANI-based comparative analyses, epidemiological tracking, and source attribution between human and swine isolates. It is therefore essential that diagnostic laboratories in newly affected or emergent regions become familiar with this pathogen. *S. suis* is a well-recognized pathogen in diagnostic laboratories for both human and swine diseases in Asia, relatively well known in Europe, but remains largely underrecognized in the Americas^8^.

Antimicrobial resistance is a growing concern for livestock services worldwide^10,11,22,29,33,34^. Use of antimicrobial agents, including not only classical antibiotics but also antimicrobial metal ions, combined with the intensive practices of the food animal industry can significantly contribute to this problem^57^. Pig farming is no exception to this pattern: the absence of vaccines against *S. suis* has led to widespread antibiotic use to control this pathogen worldwide^58^ *.* A recent review of antimicrobial resistance mechanisms in *S. suis* highlights the high global prevalence of resistance to tetracyclines, lincosamides, and macrolides^58^. However, additional resistance patterns have also been reported, including mutations affecting β-lactams—primarily through alterations in penicillin-binding proteins (PBPs)—and resistance to pleuromutilins, amphenicols, trimethoprim, aminoglycosides, glycopeptides, and quinolones^10,11,22,29,34^. Antibiotics not routinely used in human medicine, such as florfenicol, were included in this study to better reflect antimicrobial exposure within swine production environments^58^. Florfenicol is extensively employed in veterinary practice for treating respiratory and systemic infections in pigs and represents a major selective pressure for resistance development in *S. suis* populations^10,11,22,29,41^. An adaptive mechanism contributing to both virulence and antibiotic resistance in most *S. suis* strains is biofilm formation, which typically reaches maturity after approximately 60 h^58^. Biofilms confer significant resistance, particularly to β-lactams but also to quinolones, lincosamides, aminoglycosides, tetracyclines, and macrolides^58^.

Antimicrobial resistance data from human *S. suis* isolates in Brazil is limited^2,9,31,32^. Moreover, no organism-specific interpretative breakpoints exist for this species, and susceptibility assessments rely on Clinical and Laboratory Standards Institute (CLSI) criteria for viridans group streptococci^59^. This extrapolation constrains the accuracy and clinical relevance of susceptibility interpretations in human infections. Understanding antibiotic usage patterns in the swine industry allows for a more precise assessment of resistance dynamics in animal isolates and provides critical insight into cross-resistance mechanisms that could affect zoonotic transmission and treatment outcomes in humans. A long-term French study (1994–2020, n = 200) nonetheless confirmed that β-lactams remain the standard treatment for both human and swine infections^60^. Brazilian data from swine and human isolates also contribute to understanding the preserved activity of β-lactams and florfenicol^2,9,10,12,31,32^.

In Brazil, one of the world's major pork-producing countries, targeted prevention and surveillance of *S. suis* outbreaks remain scarce and unaddressed by the main national institutions responsible for swine production, such as the PAN-Br AGRO program^34^. Its increasing prevalence in humans in Brazil, and throughout Latin America^8^, highlights the urgent need for specific control measures within pig farming. Availability of resources in underserved regions, close monitoring of *S. suis* in outbreak areas, and effective containment strategies are still lacking. In Ceara, which accounts for most human cases reported in Brazilian literature, no microbiological studies on *S. suis* in swine production have been conducted. Additionally, training and education of farm personnel on biosecurity measures and disease recognition are critical components of outbreak prevention^11-19^. Ultimately, addressing this zoonosis within the scope of the PAN-Br AGRO antimicrobial resistance program is of evident importance, particularly given the propensity of certain *S. suis* strains to develop antimicrobial resistance^34,58^.

Veterinary management recommendation for swine *S. suis* outbreaks primarily involves implementing strict biosecurity measures, including controlled entry and exit of animals and personnel on farms, applying appropriate sanitary downtime, and correcting environmental predisposing factors^35,36,44,45,60^. In this context, environmental improvements are needed to mitigate risks associated with inadequate ventilation, overcrowding, and poor hygiene conditions, which contribute to the accumulation of ammonia, elevated thermal fluctuations, and ultimately create an environment favorable to pathogen establishment^11-19,29^. During clinical outbreaks, in addition to supportive symptomatic treatment, causal therapy is based on the preferential use of β-lactam antibiotics, which remain first-line drugs due to their high efficacy and low resistance rates compared with other antimicrobial classes, even against biofilm-producing strains^29,33,40,59,60^. Effective management and control of swine *S. suis* infection may contribute to mitigating future human cases and potential outbreaks.

### Limitations

Several study limitations warrant consideration here. First, the scarcity of available and standardized data limits the realization of a systematic review or meta-analysis focused on the clinical aspects of *S. suis* infection in humans or swine. Second, most existing studies focus on prevalence and serotype identification in swine, with minimal data addressing the diagnosis, treatment, and prognosis of human cases in Brazil. Third, access to molecular diagnostic methods for CNS infections across the country remains limited, which may contribute to underdiagnosis and underreporting of human cases. Counterintuitively, the improved availability of advanced diagnostic methods in several tertiary centers, such as infectious disease referral hospitals in northeastern Brazil, may have facilitated the detection of human cases via techniques like PCR and MALDI-TOF MS^53,54,56^. In the swine sector, northeastern Brazil continues to experience a significant lack of diagnostic infrastructure for detecting and managing swine diseases, including *S. suis*. Most *S. suis* isolates sequenced in South America have originated from Argentina^8^; to date, no human-derived Brazilian strains have undergone genomic sequencing. Obtaining such data would provide valuable insights into the virulence factors and epidemiological origins of Brazilian strains capable of infecting humans. Finally, human *S. suis* meningitis is not a notifiable disease in Brazil, and additional cases may have occurred without proper investigation or documentation in the medical literature.

Herein, we outline key areas for improvement to enhance awareness of this pathogen. Brazil is a continental country, and many regions—particularly in the North and Northeast—remain socioeconomically disadvantaged and have limited veterinary surveillance within the swine industry^45-47^. Additionally, clinicians should remain vigilant regarding this pathogen and the potential for misidentification, particularly when evaluating patients with acute meningitis and relevant occupational exposures,^5,6,50^. As human *S. suis* meningitis is not a notifiable disease in Brazil, additional cases may have occurred without proper investigation or documentation in the medical literature. Most laboratories do not routinely perform comprehensive phenotypic identification to accurately characterize antimicrobial susceptibility profiles and resistance patterns^5,6,53,55,59^. Genomic investigation of *S. suis* from human infections and swine outbreaks is essential to elucidate strain characteristics, virulence determinants, transmissibility, and potential sources of infection^8,56^. Strengthened surveillance of the disease in both animals and humans is imperative to improve understanding of the epidemiology, associated risk factors, and overall burden of *S. suis* infections in Brazil.

## CONCLUSION

This review highlighted the emergence of *S. suis* infection in humans and swine in Brazil. S. *suis* infection is an emerging zoonosis in the country, with recent human cases signaling a shifting epidemiological landscape and revealing substantial gaps in surveillance and diagnostic capacity. The growing industrial pig farming combined with occupational exposure and limited access to molecular tools, particularly in the Northeast, creates conditions that may favor underrecognition of both human and swine disease. Although the number of Brazilian human cases remains low and all reported infections have so far presented as CNS disease with no fatalities, the high burden of auditory sequelae underscores the clinical relevance of this pathogen. The mismatch between human case distribution and the scarcity of swine-focused studies highlights urgent structural deficiencies in veterinary diagnostics and monitoring. Strengthening a One Health approach is essential, integrating improved laboratory capacity, outbreak investigation, genomic surveillance, and targeted biosecurity measures in pig production. Broader adoption of advanced methods such as PCR, mNGS, and WGS is critical for clarifying transmission dynamics and characterizing circulating strains. Ultimately, coordinated public health and veterinary strategies are needed to mitigate disease burden, prevent future spillover, and guide national policies, including incorporation of *S. suis* into antimicrobial resistance programs.

## Data Availability

The complete anonymized dataset supporting the findings of this study is included within the article itself.
